# Partially Hydrolyzed Guar Gum Attenuates d-Galactose-Induced Oxidative Stress and Restores Gut Microbiota in Rats

**DOI:** 10.3390/ijms20194861

**Published:** 2019-09-30

**Authors:** Xiaoyan Liu, Chenxuan Wu, Dong Han, Jun Liu, Haijie Liu, Zhengqiang Jiang

**Affiliations:** Beijing Advanced Innovation Center for Food Nutrition and Human Health, College of Food Science and Nutritional Engineering, China Agricultural University, Beijing 100083, China; liuxiaoyan@cau.edu.cn (X.L.); wuchenxuan@cau.edu.cn (C.W.); donghancau@cau.edu.cn (D.H.); junliu@cau.edu.cn (J.L.)

**Keywords:** partially hydrolyzed guar gum, d-galactose, aging, oxidative damage, dysbacteriosis

## Abstract

Partially hydrolyzed guar gum (PHGG) has received considerable attention for its various bioactive functions. The injection of d-galactose can cause aging-related injury which is usually resulted from oxidative stress on tissues and cells. In this study, d-galactose (200 mg/kg/day) was injected into rats, and the protective effects of PHGG (500, 1000, and 1500 mg/kg/day) against oxidative damages, as well as its probiotic functions, were analyzed. The results showed that PHGG treatment at a concentration of 1500 mg/kg/day greatly reduced the levels of lactic acid, nitric oxide, inducible nitric oxide synthase, advanced glycation end products, and increased the telomerase activity, by 7.60%, 9.25%, 12.28%, 14.58%, and 9.01%, respectively. Moreover, PHGG significantly elevated the activities of antioxidant enzymes and decreased the content of malondialdehyde in rat serum and brain. The oxidative damage was also significantly alleviated in the liver and hippocampus and the expressions of brain-derived neurotrophic factor and choline acetyltransferase also increased. Furthermore, PHGG treatment could significantly regulated the expression of sirtuin 1, forkhead box O1, and tumor protein p53 in the hippocampus. It also increased the levels of organic acids and improved the composition of intestinal microbiota. These findings demonstrated that PHGG treatment could effectively alleviate the oxidative damage and dysbacteriosis.

## 1. Introduction

Aging is a natural process that involves the gradual loss of physiological functions, causing enhanced morbidity and mortality due to various diseases. This process is closely related to oxidative stress [1,2,3]. One prevalent theory to explain aging is the theory of the oxygen free radical, which was first proposed by Harman in 1956 [4]. This theory posits that the macromolecules (such as nucleic acids, lipids, sugars, and proteins) that make up cells and tissues are subjected to oxidative stress induced by superoxide and other free radicals. These macromolecules then undergo different degrees of oxidation, which initiates oxidative damages and ultimately leads to organ function impairment and aging [5,6]. The malfunction of the antioxidant system decreases antioxidant enzyme activity, and excessive damages from free radicals lead to further oxidative stress and the aggravation of aging. A high dose of d-galactose is converted to aldose and hydrogen peroxide by d-galactose oxidase. The products then generate reactive oxygen species through oxidative metabolism and glycosylation, leading to oxidative stress. The accumulation of oxidation products further exacerbates the oxidative damage to tissues and cells, which then accelerates the aging process [7]. Therefore, d-galactose overload has been used to establish animal models used to conduct aging related metabolic dysfunction and oxidative stress [8,9].

Dietary supplementation with prebiotics is an effective and efficient strategy to promote the growth of beneficial bacteria. The diversity and quantity of microbiota in the intestinal tract increases with age. More specifically, the number of aerobic bacteria is decreases, and the number of anaerobic bacteria increases [10]. For old people, antibiotic use, hospitalization, and *Clostridium difficile* diarrhea also increase the number of *enterococci* [11]. Prebiotics can change the composition and vitality of microbiota and promote the proliferation of beneficial bacteria, thereby effectively regulating the balance of intestinal microbiota [12]. Prebiotics also regulate body health via the intestine–brain axis, the intestine–liver axis, or other mechanisms [13]. After fermentation by intestinal microbes, prebiotics produce a variety of metabolites that can improve the energy metabolism of colon cells, promote the growth and differentiation of intestinal epithelial cells, and improve the lipid and carbohydrate metabolism in the liver [14].

Guar gum is a water-soluble dietary fiber composed of galactomannan, a component found in guar seeds. It has been widely used as a thickener and emulsion stabilizer for food [15]. Partially hydrolyzed guar gum (PHGG) is a component derived from guar gum hydrolysate. The average molecular weight of PHGG is 2.5 × 10^4^ Da, with 24.9% oligosaccharides (degree of polymerization < 7), and up to 90.6% dietary fiber [16]. PHGG cannot be degraded by the digestive enzymes in the gastrointestinal tract, and is selectively utilized and fermented by the beneficial intestinal bacteria. These bacteria produce propionic acid, butyrate, and other short-chain fatty acids (SCFAs) to modulate the pH and further stimulate the proliferation of beneficial bacteria, thus forming a positive feedback loop to generate more SCFAs in the intestine [17,18]. There is evidence showing that PHGG plays a significant role in the treatment of patients with irritable bowel syndrome [19]. It has also been found to have the positive effects of alleviating diarrhea and reducing disease risk by lowering the intestinal uptake of fat and cholesterol [20,21]. Furthermore, PHGG is effective for attenuating chronic kidney disease by protecting the intestinal barrier [22].

Currently, despite these beneficial effects of PHGG, the impact and the mechanisms of its dietary intervention on aging and microbiota have not been well investigated. In this study, we aim to comprehensively evaluate the anti-aging effect of PHGG using the rat d-galactose-induced aging model. We analyzed multiple aging biomarkers in the brain, as well as the in vivo redox state, liver histology, aging-related protein expression, and organic acids production. The composition of fecal microbiota in rats was further analyzed to investigate the correlation between the anti-aging function of PHGG and its regulation of intestinal microbiota.

## 2. Results

### 2.1. Changes in Aging Biomarkers and Antioxidant Activities

After 10 weeks of PHGG treatment, the PHGG-treated groups showed significant improvement in aging biomarkers and antioxidant activities (Table 1). Compared with the model control (MC) group, the contents of advanced glycation end products (AGEs), nitric oxide (NO), inducible nitric oxide synthase (iNOS), and lactic acid in the high-dose PHGG (H-PHGG) group were decreased by 14.58% (*p* < 0.05), 9.25% (*p* < 0.05), 12.28% (*p* < 0.05), and 7.60%, respectively, and telomerase in the medium-dose (M-PHGG) group and H-PHGG group was significantly increased by 13.66% (*p* < 0.05) and 9.01%, respectively. The levels of brain total antioxygenic capacity (T-AOC) and catalase (CAT) in the M-PHGG group were significantly increased by 44.15% and 15.02%, and malondialdehyde (MDA) was significantly decreased by 30.40% compared with the MC group (all *p* < 0.05). Moreover, the serum levels of CAT in low-, middle-, and high-dose PHGG groups were significantly increased by 31.26%, 38.55%, and 25.88%, respectively, as compared with the MC group (all *p* < 0.05).

### 2.2. Histopathological Alterations

**Figure 1:**
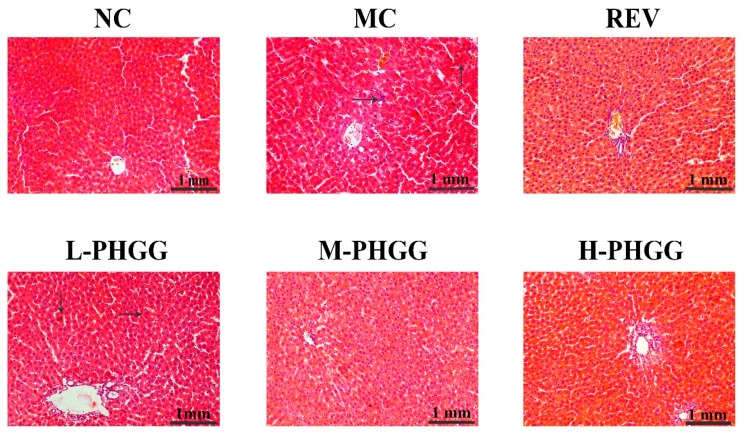
Effects of partially hydrolyzed guar gum (PHGG) on histopathological changes in the liver of rats. (Hematoxylin and eosin staining, 200×). The hepatocytes fit morphological criteria of apoptosis labeled by arrows. NC, normal control (saline); MC, d-gal model control (saline); REV, resveratrol (20 mg/kg/day); L-PHGG, 500 mg/kg/day; M-PHGG, 1000 mg/kg/day; H-PHGG, 1500 mg/kg/day.

Liver morphology was analyzed by hematoxylin and eosin (HE) staining (Figure 1). In the MC group, the histopathological examination revealed that hepatocytes were loosely arranged and enlarged. Some cells showed signs of apoptosis, and the nuclei were deeply stained and condensed. There were vacuoles of different sizes in the cytoplasm and inflammatory cell infiltration. However, after PHGG treatment, these abnormal features disappeared, and the hepatocytes became densely organized with a restored structure, exhibiting an intact cell structure, clear cell border, and normal cell size. Further, their nuclei were round and centered, and neither lipid droplets nor inflammatory cell infiltration was evident. The overall liver histomorphology was significantly improved after PHGG treatment. No noticeable difference was observed between the resveratrol (REV) group and the PHGG treatment groups.

**Figure 2:**
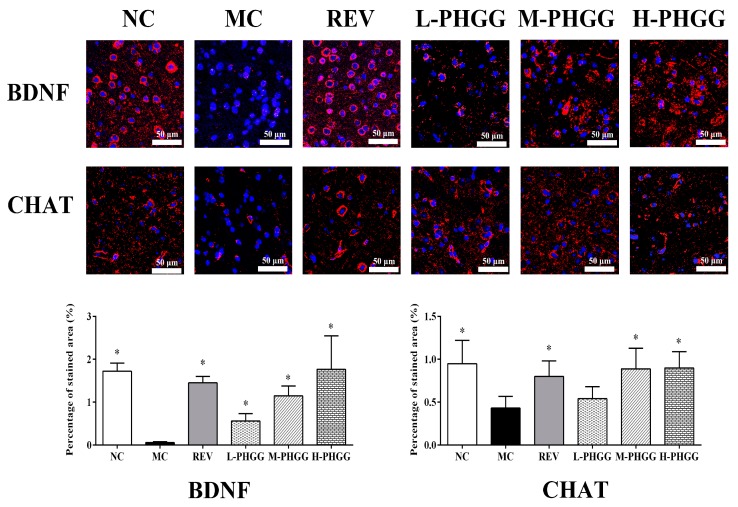
Effects of PHGG on the protein expression of brain-derived neurotrophic factor (BDNF) and choline acetyltransferase (ChAT) in the rat hippocampus. Representative images of immunofluorescence staining (600×) of BDNF and ChAT are shown. Cell nuclei are stained in blue, and BDNF/CHAT protein are stained in pink. The percentage of the BDNF-/CHAT-stained area is presented. Statistical significance between the model control group and the other groups was determined by the Kolmogorov–Smirnov test and Levene’s test for hypotheses of normality and variance homogeneity, followed by Student’s *t*-test or Mann–Whitney’s U non-parametric test. * *p* < 0.05 compared with the model control group. NC, normal control (saline); MC, d-gal model control (saline); REV, resveratrol (20 mg/kg/day); L-PHGG, 500 mg/kg/day; M-PHGG, 1000 mg/kg/day; H-PHGG, 1500 mg/kg/day.

An immunofluorescence assay was performed to examine the levels of choline acetyltransferase (ChAT) and brain-derived neurotrophic factor (BDNF) (Figure 2). Compared with the normal control (NC) group, the MC group showed significantly reduced ChAT staining and BDNF staining in the hippocampus. After the REV or PHGG treatment, the expression levels of ChAT and BDNF were greatly increased.

### 2.3. Changes in the Expression Levels of Aging Related Proteins and Genes

**Figure 3:**
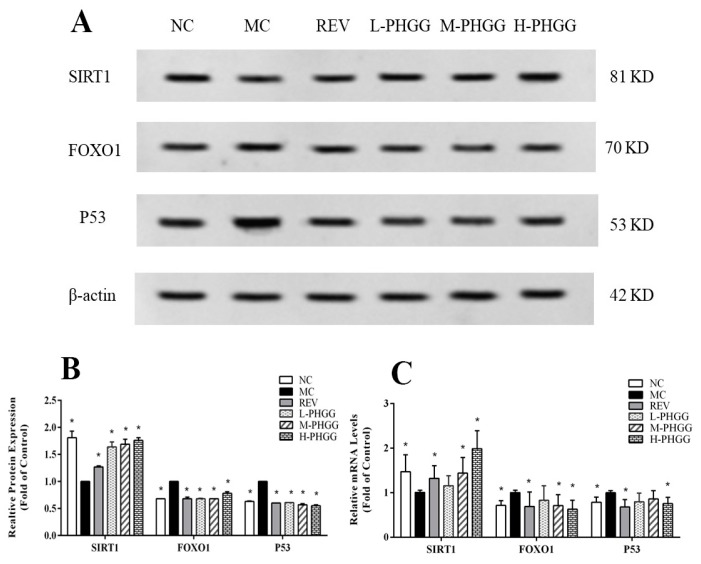
Effects of PHGG on the levels of sirtuin 1 (SIRT1), Forkhead box O 1 (FOXO1), and tumor protein P53 (P53) protein expression in the rat hippocampus. (**A**) A representative image of the Western blotting assay. (**B**) The presented data of the Western blotting assay are expressed as the fold change compared with the NC control, which was arbitrarily set to 1. (**C**) The mRNA levels of SIRT1, FOXO1 and P53 genes relative to GAPDH and normalized with the control, which was arbitrarily set to 1. Statistical significance between the model control group and the other groups was determined by the Kolmogorov–Smirnov test and Levene’s test for hypotheses of normality and variance homogeneity, followed by Student’s *t*-test or Mann–Whitney’s U non-parametric test. * *p* < 0.05 compared with the model control group. NC, normal control (saline); MC, d-gal model control (saline); REV, resveratrol (20 mg/kg/day); L-PHGG, 500 mg/kg/day; M-PHGG, 1000 mg/kg/day; H-PHGG, 1500 mg/kg/day.

Protein bands for sirtuin 1 (SIRT1), Forkhead box O 1 (FOXO1), and tumor protein P53 (P53) in brains were visualized and quantified together (Figure 3A,B). Compared with the NC group, the expression level of SIRT1 was decreased, while the expression level of FOXO1 and P53 were increased in the d-galactose induced aging group. These changes were significantly reversed after PHGG treatment. The level of SIRT1 proteins in the PHGG treatment groups was markedly higher than that in the MC group, whereas PHGG treatment decreased FOXO1 and P53 protein expression (all *p* < 0.05).

Q-PCR results showed that compared to the MC group, PHGG treatment with 1500 mg/kg/day significantly reversed the d-galactose induced decrease of SIRT1 and increase of FOXO1 and P53 (*p* < 0.05, Figure 3C). PHGG ameliorated the expression of key genes involved in the brain oxidative stress. The results indicated that the regulation effect of PHGG on these protein and genes were identical.

### 2.4. Changes in Organic Acids Concentration

**Figure 4:**
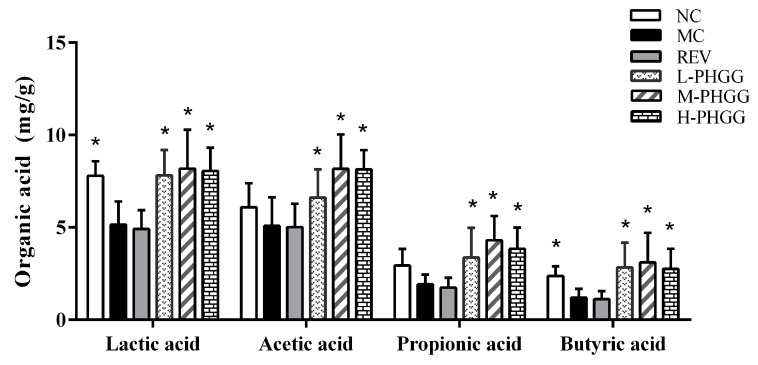
The concentration of lactic acid, acetic acid, propionic acid, and butyric acid. Statistical significance between the model control group and the other groups was determined by the Kolmogorov–Smirnov test and Levene’s test for hypotheses of normality and variance homogeneity, followed by Student’s *t*-test or Mann–Whitney’s U non-parametric test. * *p* < 0.05 compared with the model control group. NC, normal control (saline); MC, d-gal model control (saline); REV, resveratrol (20 mg/kg/day); L-PHGG, 500 mg/kg/day; M-PHGG, 1000 mg/kg/day; H-PHGG, 1500 mg/kg/day.

The organic acids content in rat feces was determined (Figure 4). After PHGG intervention, the lactic acid and SCFA contents were significantly different from those of the MC group. The concentration of acetic acid, propionic acid, butyric acid, and lactic acid in the M-PHGG group were significantly increased by 58.52%, 60.82% (*p* < 0.05), 123.95% (*p* < 0.05), and 157.02% (*p* < 0.05) compared with the MC group, respectively. This result suggests that the intake of PHGG effectively increased the production of lactic acid and SCFAs.

### 2.5. Changes in Gut Microbiota Composition

**Figure 5:**
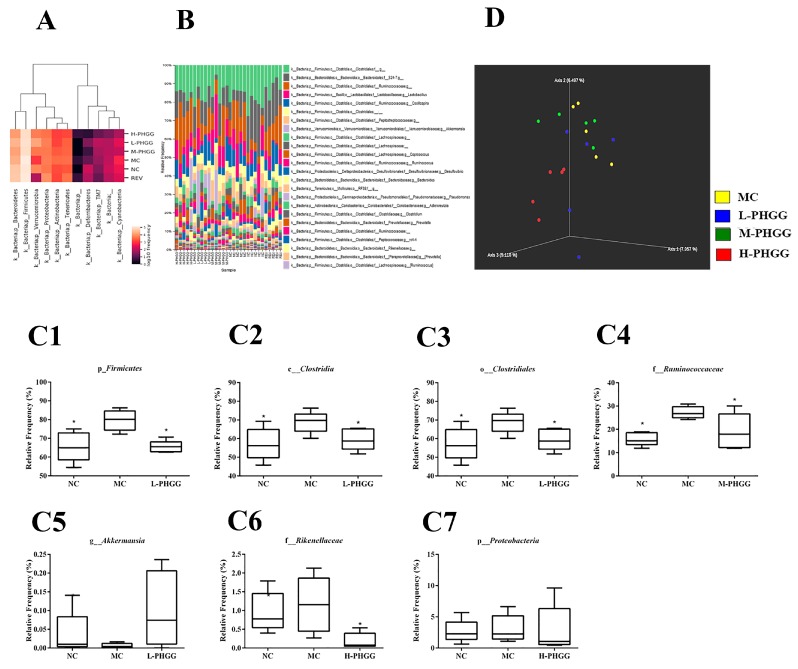
Effects of PHGG on the murine gut microbiome based using 16S rDNA sequencing. (**A**) Microbiota bacterial changes at the phylum level for each treatment groups. (**B**) Microbiota relative reference (%) are plotted to the genus taxonomy level for each fecal sample. (**C**) Bacterial population differences among the displayed groups on different taxonomy levels (p, phylum; c, class; o, order; f, family; g, genus). (**D**) Principle coordinates analysis (PCoA) of the model control and PHGG groups based on the Jaccard distance. Statistical significance between the model control group and the other groups was determined by the Kolmogorov–Smirnov test and Levene’s test for hypotheses of normality and variance homogeneity, followed by Student’s *t*-test or Mann–Whitney’s U non-parametric test. * *p* < 0.05 compared with the model control group. NC, normal control (saline); MC, d-gal model control (saline); REV, resveratrol (20 mg/kg/day); L-PHGG, 500 mg/kg/day; M-PHGG, 1000 mg/kg/day; H-PHGG, 1500 mg/kg/day.

Fecal samples were sequenced to assess the effect of PHGG on the gut microbiota structure after 10 weeks of feeding. A total of 1-,296-,350 16S rDNA raw gene sequences were obtained from 35 samples through Illumina Miseq sequencing. The average number of sequences from each sample was 43-,212. The microorganism classification at the phylum level and the cluster analysis of bacteria abundance are shown in Figure 5A. After 10 weeks of diet feeding, the increased abundance of *Verrucomicrobia* and the reduced abundance of *Firmicutes* and *Actinobacteria* in the PHGG treatment groups indicated an increase in beneficial bacteria and a reduction in detrimental bacteria, compared with the MC group. At the phylum level, the major taxonomic units include *Firmicutes*, *Bacteroidetes*, *Verrucomicrobia*, and *Proteobacteria* (Figure 5B). The relative frequency of *Firmicutes* in the PHGG treatment groups was reduced compared with that in the MC group, while *Verrucomicrobia* was increased. At the genus level, the five predominant groups identified were *Lactobacillus*, *Oscillospira*, *Akkermansia*, *Coprococcus*, and *Ruminococcus* (Figure 5B). The relative frequency of *Oscillospira* and *Coprococcus* was reduced in the PHGG treatment groups. The number of bacteria of the genus *Lactobacillus* in the phylum *Firmicutes*, as well as the genus *Bacteroides* in the phylum *Bacteroidetes*, was increased after PHGG treatment. Interestingly, compared with the MC group, the most significant changes at the phylum level, the class level, the order level and the family level were *Firmicutes*, *Clostridia*, *Clostridiales*, and *Ruminococcaceae* respectively. The relative frequency of *Firmicutes*, *Clostridia*, and *Clostridiales* was decreased by 17.71%, 13.38%, and 13.39% in the L-PHGG group, respectively (all *p* < 0.05) (Figure 5(C1–3)). The relative frequency of *Ruminococcaceae* was decreased by 29.86% in the M-PHGG group (*p* < 0.05) (Figure 5(C4)). Furthermore, the relative frequency of the genus *Akkermansia*, which is associated with beneficial bacteria was increased, while that of *Rikenellaceae* and *Proteobacteria* was reduced (Figure 5(C5–7)). The overall structure of gut microbiota in the six groups of mice was analyzed by principal coordinates analysis (PCoA) based on the Jaccard distance (Figure 5D). Similar gut microbiota structures were observed among rats in the same group. Different dosages of PHGG led to different gut microbiota structures, in particular, the H-PHGG group was very different from other groups.

## 3. Discussion

This study found that PHGG reduced the release of lactic acid and NO in the brain, inhibited the activity of iNOS, enhanced the activity of antioxidant enzymes, repaired histopathological damage, and regulated the expression of FOXO1, P53, and SIRT1. Moreover, it increased the content of organic acids in the intestine and regulated the microbiota structure.

d-galactose has been widely utilized for anti-aging research [9,23,24]. It is converted to carbon dioxide and xylulose via galactose oxidase or dehydrogenase, and it is accompanied by the excessive production of free radicals and the slow formation of stable AGEs [24]. Excessive AGEs can alter the iNOS signaling pathway and induce the synthesis of iNOS, which then leads to more NO production. When NO is excessive, it interacts with free radicals and forms the cytotoxic “free radical-peroxide nitrite” complex, leading to free radical-mediated neuronal damages and aging [25]. Rats have a complete system, which includes CAT and glutathione peroxidase (GSH-Px), to eliminate free radicals under normal conditions, in order to maintain the free radical balance [26]. An excessive supply of d-galactose can interfere with the normal glycolipid metabolism. When large amounts of glycolipids enter the mitochondria and undergo oxidative decomposition, massive levels of reactive oxygen species are produced, causing increased free radical production and reduced antioxidant capacity. The imbalance of oxygen free radical metabolism induced by d-galactose is an important cause of aging. Our results show that PHGG treatment balanced the NO content in the brain, and selectively inhibited the excessive release of iNOS (Table 1). With PHGG treatment, the level of MDA decreased, and antioxidant enzymes increased to different degrees. The changes in antioxidant enzyme activity suggest that the disorder of free radical metabolism and the oxidative damage were effectively restored and repaired, respectively, by PHGG treatment.

As is well known, the hippocampus is an important region for advanced neurological activities such as learning and memory. Compared with other organs, the brain is more sensitive to oxidative damage, which may lead to hippocampal neuronal degeneration, DNA damage, and brain aging [23]. Therefore, hippocampal neurons, especially CA1 pyramidal neurons, are widely used to study brain aging [27]. Our study indicates that the expression levels of BDNF and ChAT were increased in the PHGG treatment groups (Figure 2). These effects might be mediated by enhanced antioxidant enzyme activities, reduced oxidative damage, and increased synthesis of the neurotransmitter acetylcholine. The function of the hexanoylcholine system was thereby maintained, which eventually led to brain protection and delayed brain aging.

The SIRT1, FOXO1, and P53 genes have been shown to participate in the oxidative stress response. Transgenic mice overexpressing SIRT1 are more metabolically active and have a longer lifespan [28]. SIRT1 can activate transcription factors in the FOXO family to promote the expression of superoxide dismutase [29]. Moreover, SIRT1 can also inhibit the production of iNOS and NO by deacetylation, thus reducing the intracellular free radical load [30]. Our results indicated that SIRT1 expression was up-regulated in the PHGG treatment groups, leading to the recovery of oxidative stress resistance and the repair of body damage (Figure 3). P53 is an actively regulated protein. It inhibits cell proliferation and induces apoptosis or senescence. P53 responds to various cellular stresses, including DNA damage, oxidative stress, hypoxia, and oncogenic signaling [31]. SIRT1 can inhibit P53-mediated transcription by deacetylating the P53 promoter, thereby reducing cell apoptosis and prolonging cell life [32]. In this study, SIRT1 expression was down-regulated in the MC group. Thus, the deacetylation of the P53 promoter was attenuated and P53 expression was increased (Figure 3). PHGG treatment increased the SIRT1 level, so the enhanced deacetylation of the P53 promoter resulted in a significant decrease in P53 expression. Previous studies have shown that resveratrol can activate SIRT1, reduce P53 activity, and prolong the lifespan of mice fed a high-calorie diet [33,34]. PHGG showed an effect that was similar to the positive control drug resveratrol. It up-regulated the expression of SIRT1 and decreased the expression of FOXO1 and P53. These effects greatly alleviated the oxidative damage induced by d-galactose and protected the rats from aging.

PHGG, a component from guar gum hydrolysate, has been demonstrated to possess excellent prebiotic activity [17,18]. SCFAs are produced by intestinal microbiota. The concentration of lactic acid and SCFAs in the PHGG treatment groups were significantly increased (Figure 4). From this study, it is deduced that PHGG cannot be degraded by the digestive enzymes in the gastrointestinal tract and is selectively utilized and fermented by beneficial bacteria to produce more SCFAs. SCFAs lower the pH, inhibit the growth of detrimental bacteria that are acid sensitive, and promote the proliferation of beneficial bacteria such as *Lactobacilli* and *Bifidobacteria*. PHGG treatment not only affected the antioxidant status in vivo, but also changed the microbiota composition. The gut microbiota affects aging, and is also influenced by aging. PHGG treatment was associated with elevated levels of beneficial bacteria and reduced levels of detrimental bacteria compared with the MC group, indicating that it may have prebiotic potentials (Figure 5). The genus *Akkermansia* was increased in the L-PHGG group (Figure 5(C5)). In a previous study, it was proposed that the genus *Akkermansia,* which is associated with beneficial effects in certain diseases was significantly decreased in middle-aged mice and almost completely disappeared in old mice [10]. Furthermore, the levels of bacteria in the *Rikenellaceae* family were reduced after PHGG treatment (Figure 5(C6)). *Rikenellaceae* was previously determined to be the most significantly over-represented taxon in middle-aged and older mice [35]. In the H-PHGG treatment group in this study, *Proteobacteria* levels were decreased compared with those in the MC group (Figure 5(C7)). The *Proteobacteria* species are considered to be facultative anaerobes and opportunistic pathogens. The abundance of *Proteobacteriac* increases in the gut microbiome of older individuals, leading to increased aging-associated inflammation [36]. Furthermore, the number of bacteria of the genus *Lactobacillus* and that of the genus *Bacteroides* were enriched after PHGG treatment. The genus *Bacteroides* ferments carbohydrates and proteins and thereby reduces caloric intake. There is increasing evidence that low-calorie diets can delay aging [37,38]. Therefore, these bacteria may be responsible for the anti-aging effects of PHGG. The results of this study indicate that PHGG has a great impact on the structure of intestinal microbiota: it promotes the proliferation of beneficial bacteria and reduces the number of detrimental bacteria.

## 4. Materials and Methods

### 4.1. Materials and Animals

PHGG (molecular weight ca. 2.5 × 10^4^ Da) was obtained from Guaerrun Biotechnology Co., Ltd. (Beijing, China). d-galactose and resveratrol were purchased from Sigma-Aldrich (St. Louis, MO, USA). Primary antibodies for BDNF, ChAT, SIRT1, FOXO1, and P53 were purchased from Proteintech Group (Chicago, IL, USA). Commercial kits purchased from Beijing SINO-UK Institute of Biological Technology (Beijing, China) were used for determining T-AOC, CAT, GSH-Px, MDA, lactic acid, telomerase, NO, iNOS, and AGEs. Standards and other reagents were obtained from National Drug Reagents Co., Ltd. (Beijing, China). All other chemicals and reagents used were of analytical grade.

A total of 60 male Wistar rats were purchased from Beijing Weitong Lihua Animal Technology Co. Ltd. (Beijing, China, certificate no. SCXK (Jing) 2016-0011). The rats were 3 months old, with a mean body weight of (200 ± 20) g. All animals were raised and handled in accordance with the Guidelines for the Care and Use of Laboratory Animals [39]. All animal experiments and procedures were approved by the institutional animal care and use committee of China Agricultural University, and the approval number is 20135001-3, on 10 January 2018. The rats were housed individually in ventilated cages, and the room was kept at a constant temperature of 25 ± 5 °C and humidity of 50% ± 10%, with a 12/12 h light/dark cycle. The rats were allowed to adapt to the room conditions for 14 days before the experiments, and they had free access to food and water during this period. The experimental diet contained 100 g/kg moisture, 201 g/kg protein, 41 g/kg fat, 34 g/kg fiber, and 3.4 kcal/kg energy.

### 4.2. Grouping and Drugs Administration

The rats were randomly divided into 6 groups (10 rats per group) and treated for 10 consecutive weeks. The grouping and treatments are as follows: Normal control group (NC), model control group (MC), and resveratrol treatment group (REV) were administered via oral gavage with resveratrol at 20 mg/kg/day; and three PHGG treatment groups were administered via oral gavage with PHGG at low (500 mg/kg/day, L-PHGG), medium (1000 mg/kg/day, M-PHGG), or high dosages (1500 mg/kg/day, H-PHGG). All rats except for the NC group were injected with 200 µL of d-galactose (200 mg/kg BW) via i.h. injection every day. The body weight and the amount of consumed food and water were monitored and recorded weekly.

### 4.3. Measurement of Biochemical Indices

After the end point of the experiment, all animals were anesthetized and sacrificed. Blood samples were collected via the arteria cruralis and then centrifuged at 3500 rpm for 15 min at 4 °C. The supernatant serum was collected for biochemical assays. The hippocampus was harvested and homogenized, and 10% (*v*/*v*) hippocampus homogenate in saline was then prepared and centrifuged at 5000 rpm for 10 min at 4 °C. The supernatant was collected for various biochemical assays. The aging biomarkers in the hippocampus, including lactic acid, NO, iNOS, telomerase and AGEs, were determined according to the manufacture’s protocols. Briefly, lactic acid was measured by nicotinamide adenine dinucleotide which can convert lactic acid into pyruvic acid. NO was determined by nitrate reductase and sulfanilic acid. Telomerase was measured using the telomeric repeat amplification protocol. AGEs and iNOS were detected using an enzyme-linked immunosorbent assay kit. The antioxidative capacities in both the hippocampus and serum, including T-AOC, CAT, GSH-Px, and MDA were determined by commercially available kits. Briefly, T-AOC was measured by the reduction reaction of Fe^3+^ to Fe^2+^ and then chelated with porphyrin. CAT was determined using ammonium molybdate which rapidly terminates the degradation reaction of hydrogen peroxide catalyzed by CAT. GSH-Px activity was measured by detecting the reaction between reduced glutathione and dithibis-nitrobenzoic acid. The level of MDA was measured by the thiobarbituric acid test. All of the procedures completely complied with the manufacturer’s instructions.

### 4.4. Histopathological Examination

The rat liver tissues were fixed in 10% (*v*/*v*) buffered formalin for 48 h. The fixed samples were dehydrated using graded alcohol and then transformed into paraffin wax. Small portions with a thickness of 5 μm were selected and mounted on slides. The samples were used for staining with hematoxylin and eosin after deparaffinization to observe histopathological changes. The images were acquired by a digital camera (Nikon Digital Sight DS-Fi1c) and image acquisition software.

### 4.5. Immunofluorescence Assay

An immunofluorescence assay was used as previously described with slight modifications [40]. Namely, hippocampus tissue sections (4 µm) were permeabilized and then blocked in 5% bovine serum albumin for 20 min. After that, the tissues were incubated overnight at 4 °C with primary antibodies including those against BDNF (1:200) and ChAT (1:200). Then the tissues were incubated with fluorescein isothiocyanate (1:200) at 37 °C for 1 h. Sections were immediately examined at 200× magnification under a fluorescence microscope.

### 4.6. Western blot analysis and Quantitative Real-Time PCR

The total proteins content was extracted from hippocampus tissue, and the concentrations were measured by a bicinchoninic acid assay. The assay was used to examine SIRT1, FOXO1, and P53 expression and was performed as previously described [41]. A chemiluminescent electrochemiluminescence assay kit and the ChemiDoc XRS system (Bio-Rad, Richmond, CA, USA) were used to detect the target protein. Gray semi-quantitative analysis was performed by Quantity One software (Bio-Rad, Richmond, CA, USA). The protein bands were quantified using densitometry. Values are expressed as the fold change with respect to beta-actin and normalized with the control, which was arbitrarily set to 1.

Total RNA was isolated from brain tissues according to the manufacturer’s instructions (9767 mRNA extraction kits, Takara), followed by the reverse transcription reaction using PrimeScript™ RT Master Mix RR036A (Takara, Dalian, China). SIRT1, FOXO1, P53 and GAPDH were amplified through the LightCycler^®^ 96 real time-quantitative polymerase chain reaction (RT-qPCR) system (Roche, Mannheim, Germany). The RT-qPCR conditions were as follows: 30 s of pre-denaturation at 95 °C, followed by 40 cycles of 5 s at 95 °C, 60 °C for 30 s, and 72 °C for 30 s. The expression level of each targeted gene was quantified using the comparative method (2^−ΔΔ*C*t^), following normalization with GAPDH. The same reaction was performed in triplicate. The PCR primers are listed in Table 2.

### 4.7. Organic Acids Analysis

The organic acid concentration was measured by high performance liquid chromatography (HPLC) according to Dostal et al. with slight modification [42]. Various feces samples (200 mg per sample) were homogenized with 0.8 mL of concentrated sulfuric acid. After centrifugation, the supernatants were diluted before injection. HPLC was performed on an Agilent Hi-Plex machine (250 × 4.6 mm) at a flow rate of 0.3 mL/min at 55 °C. Sulfuric acid (5 Mm) was used as the eluent solution. The contents of acetic acid, propionic acid, butyric acid and lactic acid contents were used as standards.

### 4.8. Intestinal Microbiota Analysis

The intestinal microbiota composition of the rats was also analyzed. Upon sacrifice, mice ceca were immediately collected and frozen at −80 °C. Genomic DNA extraction was carried out using the Qiagen DNeasy kit. DNA obtained from each sample was diluted to the same concentration (10 nmol/l) and then subjected to PCR amplification using primer pair targeting the V3-V4 region (338F 5′-ACT CCT ACG GGA GGC AGC A-3′ and 806R 5′-GGA CTA CHV GGG TWT CTA AT-3′) to generate an amplicon of 480 bp. Illumina MiSeq-PE250 system was employed for the paired-end amplicon sequencing according to manufacturer’s recommendations. Paired-end raw sequencing data (fastq. files with quality scores) were de-multiplexed, joined, filtered, analyzed, and visualized using the QIIME2 pipeline (https://qiime2.org/; version 2018.08). Trimmed raw sequences were joined and denoised by the DADA2 package [43]. Taxonomy of each sample was established according to sequence variants [44] assigned to a Greengenes 13_8 [45] trained Naïve Bayes classifier [46] using a q2-feature-classifier [47]. A heatmap that represents the feature table with taxonomic annotations at the phylum level was generated by q2-feature-table. Principle coordinates analysis (PCoA) plots based on Jaccard distance were generated using a q2-diversity plugin and visualized using Emperor.

### 4.9. Statistical Analysis

Data were expressed as the mean ± standard deviation (SD). Statistical significance between the model control group and the other groups was determined by the Kolmogorov–Smirnov test and Levene’s test for hypotheses of normality and variance homogeneity, followed by Student’s *t*-test or Mann–Whitney’s U non-parametric test. * *p* < 0.05 compared with the model control group was considered as statistically significant.

## 5. Conclusions

PHGG treatment showed protective effects in the form of oxidative damage repair in an aging model: It ameliorated multiple aging biomarkers, enhanced the activity of antioxidant enzymes, and repaired the histopathological damage caused by free radicals. It also regulated the expression of FOXO1 and P53 by inhibiting SIRT1. Moreover, PHGG increased the content of organic acids in the intestine and regulated the microbiota structure. Overall, PHGG treatment effectively alleviated the damage and dysbacteriosis caused by d-galactose, providing a scientific foundation for developing PHGG as an anti-aging food material.

## Figures and Tables

**Table 1:** Aging biomarkers in the brain and levels of antioxidant activity in the serum and brain.

	NC	MC	REV	L-PHGG	M-PHGG	H-PHGG
Aging biomarkers						
AGEs (ng/gprot)	394.53 ± 37.12 *	586.00 ± 71.97	429.70 ± 52.90 *	527.98 ± 91.13	533.05 ± 66.92	500.54 ± 56.71 *
NO (µmol/gprot)	0.81 ± 0.05 *	1.08 ± 0.04	0.94 ± 0.07 *	1.06 ± 0.08	1.02 ± 0.08 *	0.98 ± 0.12 *
iNOS (U/mgprot)	0.45 ± 0.06 *	0.57 ± 0.07	0.43 ± 0.07 *	0.51 ± 0.05	0.50 ± 0.04 *	0.50 ± 0.05 *
Lactic acid (µmol/gprot)	14.15 ± 1.64 *	21.96 ± 1.56	15.83 ± 2.63 *	20.59 ± 2.19	21.46 ± 2.19	20.29 ± 1.79 *
Telomerase (nU/mgprot)	103.92 ± 9.96 *	77.79 ± 7.58	104.21 ± 7.81 *	87.37 ± 6.82 *	88.42 ± 10.88 *	84.80 ± 7.62
Antioxidant activity in brain						
T-AOC (U/mgprot)	3.69 ± 0.24 *	3.42 ± 0.32	4.71 ± 0.28 *	3.69 ± 0.28	4.93 ± 0.43 *	4.66 ± 0.37 *
CAT (U/mgprot)	18.33 ± 1.69	17.57 ± 2.35	22.37 ± 1.30 *	19.73 ± 2.38	20.21 ± 2.62 *	19.22 ± 2.99
GSH-Px (U/mgprot)	536.63 ± 21.36 *	517.14 ± 19.56	612.94 ± 16.54 *	530.44 ± 27.18	534.42 ± 31.41	526.26 ± 19.01
MDA (nmol/mgprot)	1.12 ± 0.21	1.25 ± 0.32	0.87 ± 0.11 *	1.07 ± 0.34	0.87 ± 0.17 *	0.95 ± 0.11 *
Antioxidant activity in serum						
T-AOC (U/mL)	9.5 ± 2.10	8.52 ± 0.81	10.36 ± 1.05 *	8.74 ± 0.96	9.66 ± 0.83 *	8.7 ± 0.78
CAT (U/mL)	24.90 ± 9.56	18.78 ± 1.37	29.16 ± 14.8	24.65 ± 6.48 *	26.02 ± 6.58 *	23.64 ± 6.25 *
GSH-Px (U/mL)	848.95 ± 73.77	857.93 ± 86.69	895.55 ± 124.19	864.45 ± 50.10	852.97 ± 55.23	801.00 ± 34.12
MDA (nmol/mL)	2.97 ± 0.88	4.07 ± 1.57	2.29 ± 0.21 *	3.10 ± 0.40	2.94 ± 0.31 *	3.12 ± 0.56

NC, normal control (saline); MC, d-gal model control (saline); REV, resveratrol (20 mg/kg/day); L-PHGG, 500 mg/kg/day; M-PHGG, 1000 mg/kg/day; H-PHGG, 1500 mg/kg/day. AGEs, advanced glycation end products; NO, nitric oxide; iNOS, inducible nitric oxide synthase; T-AOC, total antioxygenic capacity; CAT, catalase; GSH-Px, glutathione peroxidase; MDA, malondialdehyde. Mean ± SD (*n* = 10). Statistical significance between the model control group and the other groups was determined by the Kolmogorov–Smirnov test and Levene’s test for hypotheses of normality and variance homogeneity, followed by Student’s *t-*test or Mann–Whitney’s U non-parametric test. * *p* < 0.05 compared with the model control group.

**Table 2:** Primer sequences used for semi-quantitative RT-qPCR analysis.

	Forward Primer	Reverse Primer
SIRT 1	GAAAATGCTGGCCTAATAGACTTG	TGGTACAAACAAGTATTGATTACCG
FOXO1	TCCCACACAGTGTCAAGACTACAA	CTGCTGTCAGACAATCTGAAGGA
P53	AACTTACCAAGGCAACTATG	CTTGTAGATGGCCATGGCAC
GAPDH	CTTCTTGTGCAGTGCCAGCC	CAAGAGAAGGCAGCCCTGGT

SIRT1: Sirtuin 1; FOXO1: Forkhead box O 1; P53: tumor protein p53.
